# The effect of information about the benefits and harms of mammography on women’s decision-making: study protocol for a randomized controlled trial

**DOI:** 10.1186/s13063-017-2161-7

**Published:** 2017-09-12

**Authors:** Misericòrdia Carles, Montserrat Martínez-Alonso, Anna Pons, Maria José Pérez-Lacasta, Lilisbeth Perestelo-Pérez, Maria Sala, Carmen Vidal, Montse Garcia, Ana Toledo-Chávarri, Núria Codern, Maria Feijoo-Cid, Anabel Romero, Roger Pla, Jorge Soler-González, Xavier Castells, Montserrat Rué, on behalf of the InforMa Group, Àngels Cardona, Núria Codern, Lilisbeth Perestelo-Pérez, Ana Toledo, Maria Feijoo, Montse Garcia, Carmen Vidal, Sara Buil, Clara Viñals, Laia Viñals, Montserrat Martínez-Alonso, Marta Ortega, Sandra Pla, Anna Pons-Rodríguez, Montserrat Rué, Jorge Soler, Misericòrdia Carles, Maria José Pérez, Roger Pla, Andrea Burón, Xavier Castells, Anabel Romero, Maria Sala

**Affiliations:** 1 Department of Economics, University Rovira i Virgili, Reus, Spain; 2 Research Group on Economic Evaluation and Health (GRAES), Reus, Spain; 3 Research Centre on Industrial and Public Economics, (CREIP), Reus, Spain; 4 Department of Basic Medical Sciences, University of Lleida-IRBLLEIDA, Lleida, Spain; 5 Lleida Biomedical Research Institute (IRBLLEIDA), Lleida, Spain; 6 Evaluation Unit of the Canary Islands Health Service (SESCS), Tenerife, Spain; 7 Center for Biomedical Research of the Canary Islands (CIBICAN), Tenerife, Spain; 8 Health Services Research on Chronic Patients Network (REDISSEC), Madrid, Spain; 9 Epidemiology and Evaluation Department, Hospital del Mar, Barcelona, Spain; 10 IMIM (Hospital del Mar Medical Research Institute), Barcelona, Spain; 11 Cancer Prevention and Control Program, Catalan Institute of Oncology-IDIBELL, L’Hospitalet de Llobregat, Spain; 12 Canary Islands Foundation of Health Research (FUNCANIS), Tenerife, Spain; 13 ÀreaQ, Evaluation and Qualitative Research, Barcelona, Spain; 14 Nursing and Occupational Therapy School (EUIT), Terrassa, Universitat Autònoma de Barcelona, Barcelona, Spain; 15 Department of Nursing, Faculty of Medicine, Universitat Autònoma de Barcelona, Bellaterra, Spain; 16 Catalan Health Service, Tarragona Region, Tarragona, Spain; 17 Medical School, University Rovira i Virgili, Reus, Spain; 18 Department of Medicine, University of Lleida, Lleida, Spain

**Keywords:** Screening, Breast cancer, Informed choice, Early detection, Decision aids

## Abstract

**Background:**

The decision to participate or not in breast cancer screening is complex due to the trade-off between the expected benefit of breast cancer mortality reduction and the major harm of overdiagnosis. It seems ethically necessary to inform women so that they can actively participate in decision-making and make an informed choice based on their values and preferences.

The objective of this study is to assess the effects of receiving information about the benefits and harms of screening on decision-making, in women approaching the age of invitation to mammography screening.

**Methods:**

A two-stage, randomized controlled trial (RCT). In the first stage, 40 Basic Health Areas (BHAs) will be selected and randomized to intervention or control. In the second stage, women within each BHA will be randomly selected (*n* = 400). Four breast cancer screening programs (BCSPs) of the Spanish public health system, three in Catalonia and one in the Canary Islands will participate in the study. Women in the intervention arm will receive a leaflet with detailed information on the benefits and harms of screening using mammography. Women in the control arm will receive a standard leaflet that does not mention harms and recommends accepting the invitation to participate in the biennial examinations of the BCSP.

The primary outcome is informed choice, a dichotomous variable that combines knowledge, attitudes, and intentions. Secondary outcomes include decisional conflict; confidence in the decision made; anxiety about screening participation; worry about breast cancer; anticipated regret; time perspective; perceived importance of benefits/harms of screening; perceived risk of breast cancer; and leaflet acceptability. Primary and secondary outcomes are assessed 2–3 weeks after the intervention.

**Discussion:**

This is the first RCT that assesses the effect of informing about the benefits and harms of breast cancer screening in Spain in women facing the decision to be screened using mammography. It aims to assess the impact of information on several decisional outcomes and to contribute to paving the road towards shared decision-making in breast cancer screening in our country.

**Trial registration:**

ClinicalTrials.gov registry, ID: NCT03046004. Retrospectively registered on 4 February 2017. Trial name: InforMa study.

**Electronic supplementary material:**

The online version of this article (doi:10.1186/s13063-017-2161-7) contains supplementary material, which is available to authorized users.

## Background

### Background and rationale

The objective of breast cancer screening is to find cancer at an early stage when it may be easier to treat. Evidence shows that screening reduces breast cancer mortality by around 20% [1]. However, screening can produce harms or undesired effects such as false-negative and false-positive results, and the overdiagnosis of tumors that would not cause health problems during the woman’s lifetime in the absence of screening [1].

The decision to participate or not in breast cancer screening is complex. On the one hand, there is a trade-off between the expected benefit of breast cancer mortality reduction and the major harm of overdiagnosis. On the other hand, there is uncertainty on the magnitude of overdiagnosis, with an estimated frequency of 11% of breast cancer cases from a population perspective, and about 19% from the perspective of a woman invited to screening [1]. It seems ethically necessary to inform women so that they can actively participate in decision-making and make an informed choice based on their values and preferences.

The need to develop and test decision aids (DAs) for breast cancer screening is clear. DAs are tools that help people become involved in decision-making by making explicit the decision that needs to be made, providing information about the options and outcomes, and by clarifying personal values [2]. Information on cancer screening is often biased, incomplete and persuasive [3, 4]. Many health professionals think that informing women about screening harms may deter them from being screened, with negative consequences for them [5]. A systematic review by Hoffmann and Del Mar shows that women overestimate the benefits and underestimate the harms of screening [6]. In addition, women overestimate the risk of breast cancer and most of them have not been informed of the adverse effects of screening. This explains why the majority of women believe very strongly that screening is almost always a good idea and that finding cancer early saves lives [7].

We performed a systematic review of the effects of DAs about breast cancer screening in women aged 50 years or younger, facing the decision to be screened [8]. Only four studies were included in the review [9–12], three of them published in 2015, which can be explained by the recent development of DAs for breast cancer screening. Moreover, the DAs were designed in Australia, the USA, and Germany, limiting the generalizability of the results to our country, Spain. The meta-analysis of these studies showed that DAs improve knowledge and promote informed decisions, but divergent results on decisional conflict and confidence in the decision were observed. We concluded that more research is needed for the improvement of DAs and for assessing its impact on women’s decisions.

Prior to the start of the randomized controlled trial (RCT) we performed a qualitative study with focus groups of women and health professionals with the aim of testing and improving an informative leaflet for breast cancer screening [5]. The effect of this leaflet in women facing the decision to participate in breast cancer screening is being assessed in the on-going study described by the present protocol.

### Objective

The objective of this study is to assess the effect of receiving information about the benefits and harms of breast cancer screening on informed choice and other decision-making outcomes in women approaching the age of invitation to mammography screening.

### Trial design

The study is designed as a two-stage RCT. In order to reduce intragroup contamination, in the first stage, 40 Basic Health Areas (BHAs) from two regions of Spain are selected and randomized to intervention or control. BHAs are the elementary territorial units of the health care system where primary health care and health promotion actions are provided to the population. In the second stage, women within each BHA will be randomly selected. To the best of our knowledge this is the first multicenter RCT on the effect of providing balanced information for breast cancer screening in Spain prior to being invited to screening using mammography. The primary outcome is informed choice, a dichotomous outcome that combines knowledge with consistent attitudes and intentions [12].

A pilot study was carried out with 30 women to test the recruitment procedures and the data collection process. Questionnaires were also checked for understanding and acceptability.

This study protocol follows the Standard Protocol Items: Recommendations for Interventional Trials (SPIRIT) guidelines (see Additional file 1).

## Methods

### Participants, interventions, and outcomes

#### Study setting

In Spain the National Health System provides universal and free health coverage, including early detection of breast cancer. All women resident in Spain aged 50 to 69 years are actively invited to participate in the population-based screening program by a written letter every 2 years. Breast cancer screening follows the European Guidelines for Quality Assurance in Mammographic Screening [13] and its results meet the required standards. The invitation letter contains basic information about the program, mostly about its benefits.

Four breast cancer screening programs (BCSPs) of the Spanish public health system, three in Catalonia and one in the Canary Islands will participate in the study. All the BHAs of the participating BCSPs will be stratified by socioeconomic level. For two of the BCSPs in Catalonia the strata will be quintiles of a deprivation index. For the third BCSP in Catalonia we performed a principal components analysis that indicated that immigration rate and percentage of population older than 64 years were the variables that better separated the BHAs. Based on these two dichotomized variables four strata will be obtained. For the Canary Islands, the BHA strata will be obtained considering the immigrant rate, the proximity to health care services, and a rural/urban categorical variable.

Five blocks of two BHAs (one intervention and one control) will be randomly selected from the socioeconomic strata in each BCSP, for a total of 40 BHAs. Random samples of 30 to 50 women will be obtained within each BHA. The size of the selected samples will depend on the number of eligible women in each BHA and on the observed participation in the pilot study. Women potentially eligible within the selected BHAs range from 15 to more than 300. If a selected BHA has less than 30 potentially eligible women, all the potentially eligible women of the nearest BHA within the same socioeconomic strata will be added before selection of the random sample. The target sample size is 10 women per BHA, a total of 400 women, 200 in the intervention and 200 in the control group.

### Eligibility criteria

Inclusion criteria are being a woman aged 49–50 years who, in 2–4 months, will be invited to participate in the screening program for the first time. The participant BCSPs are managed by Hospital del Mar in Barcelona, the Cancer Prevention and Control Program of the Catalan Institute of Oncology, the Canary Islands Health Service, and the Lleida Health Region.

Women will be excluded if they have a personal history of breast cancer; have difficulty speaking Spanish or Catalan; and have cognitive impairment that prevents them from understanding or completing the materials based on the interviewer’s judgment. Women with a low literacy level will not be excluded.

### Interventions

Women in the intervention arm will receive a leaflet with detailed information on the benefits (breast cancer mortality reduction, less intensive treatments) and harms (false-positive results and overdiagnosis) of screening. Women in the control arm will receive a standard leaflet that does not mention harms and recommends accepting the invitation to participate in the biennial examinations of the BCSP. The intervention leaflet is included in Additional file 2.

The intervention leaflet was drafted from existing published leaflets and its estimates of the effects of screening are drawn from published systematic reviews, clinical trials or observational studies. The leaflet was evaluated using a qualitative design with focus groups of women and health professionals [5]. We analyzed women’s perception of the leaflet information on benefits and harms of screening, and also health care professionals’ perceptions of the convenience of providing it. The leaflet was rewritten accordingly, and assessed for acceptability in a pilot study.

### Outcomes

With the aim of comparability, the outcome measures follow the Hersch et al. study protocol [14] very closely and will be collected using two questionnaires (pre-intervention (Q1) and post-intervention (Q2)) which are included in Additional files 3 and 4, respectively. Most of the outcome measures will be obtained through validated scales that have shown suitability in previous studies. We have translated them to Catalan and Spanish. The post-intervention measures will be assessed at 2–4 weeks after the intervention.

### Primary outcome

The primary outcome is informed choice. It is a dichotomous outcome defined as adequate knowledge and intentions consistent with attitudes (positive or negative) [12, 15–17]. It is included in Sections 2, 3, and 4 of the post-intervention survey. The proportion of women with an informed choice will be compared between the two study groups. The three component variables of informed choice: knowledge, attitudes, and intentions, will be analyzed and reported separately.

### Knowledge

Conceptual and numerical knowledge will be assessed following the Hersch et al. study [12] adapted to the mortality, incidence, and outcomes of screening data of our setting. The threshold to define adequate knowledge for informed choice is to score at least 50% of the available marks, including at least one numerical mark, on all the three screening outcome subscales that refer to mortality reduction, overdiagnosis, and false positives.

### Screening attitudes

Screening attitudes will be measured using five items adapted from Dormandy et al. [18]. Each item ranges from *strongly negative* to *strongly positive* [17]. Total scores can range from 5 to 25. A positive attitude is defined as a total score ≥ 20.

### Screening intentions

Intentions to participate in screening will be measured with one question with five responses that will be dichotomized as categories 1–2 (responses *definitely will* and *will*) indicating “intending” to screen and categories 3–5 (responses *unsure*, *will not,* and *definitely will not*), indicating “not intending” to screen [19, 20].

### Secondary outcomes

Secondary outcomes include decisional conflict; confidence in the decision made; anxiety about screening participation; worry about breast cancer; anticipated regret; time perspective; perceived importance of benefits/harms of screening; perceived risk of breast cancer; and leaflet acceptability. Secondary outcomes are included in Sections 5 to 7 of the post-intervention questionnaire. In addition, screening participation and reasons for non-participation will be assessed.

### Decisional process (conflict and confidence)

Decision conflict will be assessed using the Decisional Conflict Scale (10-item low-literacy version) by O’Connor [21, 22]. The items’ responses are *yes*, *no*, and *not sure*. Confidence in the decision made will be measured with three questions with five response options ranging from *very little* to *very much*.

### Anxiety about screening participation

Anxiety will be measured with the six-item short form of the Spielberger State-Trait Anxiety Inventory [23] with four responses from *not at all* to *a lot*.

### Worry about breast cancer

A single item will measure the women’s level of worry about developing breast cancer, using four verbal response categories ranging from *not worried at all* to *very worried* [16, 17].

### Anticipated regret

This will be measured with two items from a validated scale. The first measures inaction regret and the second action regret. Both items have five response categories ranging from *strongly agree* to *strongly disagree* [24, 25].

### Time perspective

This will be assessed using the short form of the Consideration of Future Consequences Scale [26]. It includes four-items with five response categories ranging from *strongly agree* to *strongly disagree*.

### Perceived importance of screening benefits/harms

This will be assessed with three questions on the importance of avoiding a breast cancer death, being diagnosed and treated for a cancer that is not harmful, and having a false-positive result, when deciding screening participation [12]. The response options range from *very important* to *not at all important*.

### Perceived personal risk of breast cancer

This will be measured with two questions, one about the perception of personal risk for developing breast cancer during lifetime, in absolute terms (with four response categories ranging from *no chance* to *high chance*) [12, 25] and relative to an average woman of the same age (using five response categories ranging from *much lower* to *much higher*) [12, 27].

### Perceived personal chances of screening benefits/harms

This will be measured with three questions on the perceived personal chance of experiencing specific outcomes if they participate in screening, compared with an average screened woman [12, 28]. The response categories range from *much lower* to *much higher*.

### Leaflet assessment

Both leaflets, intervention and control, will be assessed with five items, extracted from the nine items of Hersch et al. [12]: (1) Read the leaflet all the way through? (yes/no), (2) Length of the leaflet (responses range from *much too short* to *much too long*), (3) Balance of the leaflet (responses range from *clearly slanted towards screening* to *clearly slanted away from screening*), (4) The leaflet was clear and easy to understand (responses range from *strongly agree* to *strongly disagree*), and (5) Found the leaflet helpful in making decision (responses range from *strongly agree* to *strongly disagree*).

### Screening participation

After women have been invited to undergo the screening mammogram, participation and reasons for non-participation will be obtained through the corresponding BCSPs. Reasons for non-participation will be categorized as: already had a mammogram elsewhere; did not receive invitation; on holiday; ill/hospitalized; postponed to next round; forgot appointment; already having treatment for cancer; not interested; screening does not work; screening has adverse effects; other.

### Participant timeline

**Fig. 1:**
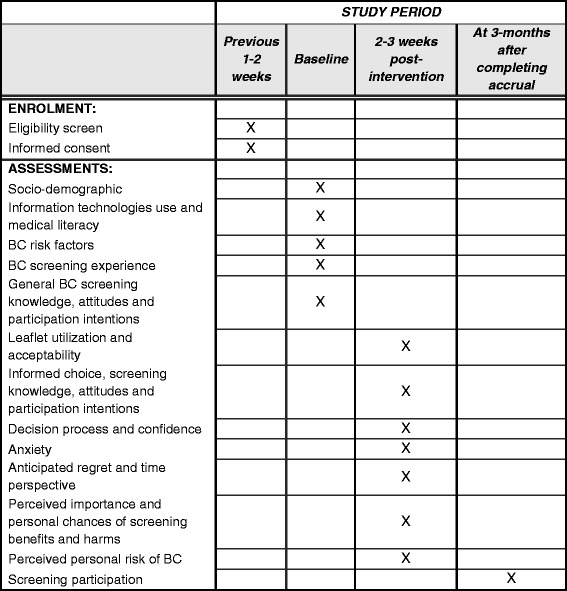
Summary of enrollment, interventions, variable assessments, and timing for measurements (SPIRIT Figure). Allocation is established at the beginning of the study

The study time period is 1 July 2016 to 31 August 2017. Allocation of the BHAs to the intervention or control groups was established at the beginning of the study. The participant timeline is detailed in Fig. 1.

### Sample size

The primary analysis will compare the proportion of women who make an informed choice in the two groups using a chi-square test and the 95% confidence interval of the difference of proportions. We have considered clinically relevant an absolute difference of 20%. Assuming that the proportion of one group is 50% (conservative scenario) and estimating an intraclass correlation coefficient equal to 0.1 (cluster sampling), in order to achieve an 80% power to detect a group difference of 20%, with a two-sided significance level of 5%, a sample size of 200 women per group is required. The 400 women will be distributed in 100 per each BCSP. This sample size is sufficient to detect a difference of 20% in the secondary outcome intention to participate and a mean difference of 0.35 standard deviations in the knowledge and attitudes scales. Assuming that 60% of women invited to participate will accept and 20% will be lost to follow-up, a minimum of 840 women, 210 per BCSP, will be invited.

### Recruitment

All selected women will receive a mailed invitation letter with a summary of the study objectives. In an interval of 2 weeks, a phone call will be made where trained interviewers briefly introduce and describe the study and determine eligibility (assessment of the inclusion/exclusion criteria). The interviewers will inform the women that participation consists of answering two questionnaires, either via web or by phone with a prior mailing of the questionnaires. Consent will be obtained orally and women will be told they can leave the study at any time, with no effect on being invited to the screening program.

## Assignment of interventions

### Allocation

#### Sequence generation, allocation concealment and implementation

In order to reduce the intragroup contamination, the first stage of sampling will randomly assign 10 BHAs of each screening program to the intervention and control groups. In total, 20 BHAs will be assigned to each study group using computer-generated blocks of size 2. In the second stage, the public system health registries will be used to select the target population using a computer-generated sequence of random numbers.

The random allocation sequence will be generated by a statistician with no contact with the participants (MR). The interviewers responsible for the recruitment of participants will not be aware of the women’s allocation.

### Blinding

It will not be possible to blind the intervention to the interviewers and participants. The interviewers will be independent of the research team and, for women who choose answering by phone, the interviews will follow a structured outline and will be continuously monitored by the study team. Statistical analyses and drawing of conclusions from these will be conducted on an intention-to-treat basis and according to the study protocol. The data analysts will be blinded to intervention status.

### Data collection, management, and analysis

#### Data collection methods

After acceptance, the first questionnaire (Q1) will be sent to the study participants. Q1 is included in the Additional file 3 and has the following sections: (1) sociodemographic data (including education level and work situation), (2) previous screening experience (opportunistic screening use, if applicable, periodicity of mammographic examinations, previous breast lesions, previous diagnostic tests for breast lesions), (3) risk factors of breast cancer (age at menarche, reproductive history, age at menopause, if applicable, use of hormone replacement therapy, use of oral contraceptive drugs, and family history of breast cancer), (4) use of information technologies, (5) general screening knowledge, (6) screening attitudes, (7) screening intentions, and (8) medical literacy according to the short version of the European Health Literacy Survey Questionnaire (HLS-EU-Q16) [29].

The post-intervention questionnaire (Q2) has the following sections: (1) leaflet acceptability, (2) screening knowledge, (3) screening attitudes, (4) screening intentions, (5) decision process, (6) decisional confidence, (7) anxiety, (8) anticipated regret, (9) time perspective, (10) perceived importance of benefits and harms, (11) perceived personal chances of screening benefits and harms, and (12) perceived personal risk of breast cancer. Participation in the screening program will be assessed at 3 months after completing accrual.

### Data management

All the data will be entered and recorded with the open source LimeSurvey [30]. Range checks and error alerts will be used to prevent invalid data. R software will be used for data analysis [31].

### Statistical methods

Primary and secondary outcomes in the two study groups will be compared using the chi-square test for categorical variables and the Student’s *t* and Mann-Whitney *U* tests for quantitative variables. These standard tests will be adjusted for the inflation in variance that can be attributed to clustering of responses within BHAs [32]. Multivariate mixed-effects models [33] will be used to assess the effect of the intervention on the outcomes accounting for women’s characteristics and the intracluster correlation induced by the BHAs. If missing values reduce the sample size by more than 10%, multiple imputation and sensitivity analysis will be used. No subgroup analyses will be performed.

## Discussion

This is the first RCT trial assessing the effect of informing about the benefits and harms of breast cancer screening in Spain in women facing the decision to be screened using mammography. The leaflet used in the intervention arm is based on current evidence from systematic reviews and has been adapted to local data on incidence and mortality.

In the qualitative study designed for developing the leaflet of the intervention group, women showed a lack of knowledge of the existence of overdiagnosis and surprise that they had not been informed of this issue [5]. The current study aims to assess the impact of informing Spanish women on several decisional outcomes and contribute to paving the road towards shared decision-making in breast cancer screening in our country.

With the aim of comparability, the outcome measures follow very closely the Hersch et al. study protocol [14]. Most of the outcome measures are obtained through validated scales that have been used in previous studies.

One of the study limitations is that the intervention may not reach women with low literacy or who are recent immigrants. Moreover, the intervention leaflet may not meet the needs of some population groups, such as people with cognitive impairment, to understand or complete the materials or persons who do not speak Spanish or Catalan.

### Trial status

As of 30 June 2017, we have enrolled 335 of our target 400 participants in the study.

## Additional files

Additional file 1: SPIRIT Checklist. Checklist of the Standard Protocol Items: Recommendations for Interventional Trials (SPIRIT) guidelines. (PDF 99 kb)

Additional file 2: Study leaflet. Leaflet with detailed information on the benefits and harms of screening with mammography, used in the intervention arm of the study. (PDF 791 kb)

Additional file 3: Pre-intervention questionnaire (Q1). Baseline characteristics of participants. (PDF 253 kb)

Additional file 4: Post-intervention questionnaire (Q2). Study outcomes. (PDF 298 kb)

## Abbreviations

BCSP: Breast cancer screening program
BHA: Basic Health Area
DA: Decision aids
HLS-EU-Q16: European Health Literacy Survey Questionnaire-short version
ICMJE: International Committee of Medical Journal Editors
ID: Identifier
Q1: Pre-intervention questionnaire
Q2: Post-intervention questionnaire
RCT: Randomized controlled trial
