# Supplementary material for: The effect of information about the benefits and harms of mammography on women’s decision-making: study protocol for a randomized controlled trial
Source: Trials. 2017 Sep 12;18:426. doi: 10.1186/s13063-017-2161-7 (PMC5596499; doi:10.1186/s13063-017-2161-7)
Supplement: Supplementary file 2 — Study leaflet. Leaflet with detailed information on the benefits and harms of screening with mammography, used in the intervention arm of the study. (PDF 791 kb) [file 13063_2017_2161_MOESM2_ESM.pdf]

NO OLVIDES QUE...

La mamografía no evita que tengas cáncer de mama. Además, no es un método perfecto; algunos tumores son muy difíciles de ver en una mamografía.

Puede ser que no tengas cáncer. Pero si lo tuvieras, el diagnóstico y tratamiento en una fase inicial del tumor puede suponer una mayor probabilidad de supervivencia.

Aunque te hayas hecho una mamografía recientemente, es importante que si notas algún cambio en el pecho vayas al médico.

La información presentada en este folleto se ha basado en artículos científicos y materiales desarrollados por el Programa de Cribado de Cáncer de Mama del National Health Service en Inglaterra, por la Colaboración Cochrane y por programas de cribado de Cataluña.

GOBIERNO DE ESPAÑA
MINISTERIO DE ECONOMÍA Y COMPETITIVIDAD
Instituto de Salud Carlos III
Unión Europea
Fondo Europeo de Desarrollo Regional
"Una manera de hacer Europa"

Estudio PI14/00113 Participación de las mujeres en las decisiones y estrategias de detección precoz del cáncer de mama. Co-financiado por el Instituto de Salud Carlos III y fondos FEDER de la Unión Europea. Participan: Institut de Recerca Biomèdica de Lleida-Universitat de Lleida, Universitat Rovira i Virgili, Institut Català d'Oncologia, Hospital del Mar y Servicio Canario de Salud.

BENEFICIOS Y EFECTOS ADVERSOS A LARGO PLAZO DE LA DETECCIÓN PRECOZ DEL CÁNCER DE MAMA

Si un grupo de 200 mujeres entre 50 y 69 años se hacen mamografías de cribado cada 2 años, cuando cumplan 80 años...

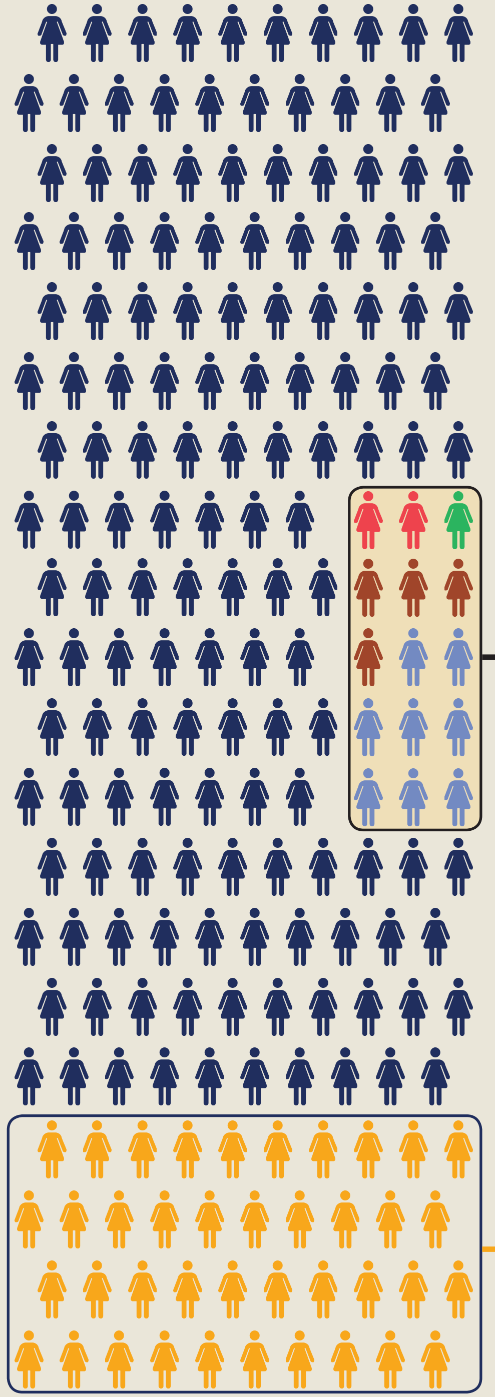

15 mujeres habrán sido diagnosticadas de cáncer de mama

BENEFICIOS DEL CRIBADO

1 sobrevivirá al cáncer de mama

Habría fallecido sin el cribado

Los tratamientos de algunas mujeres pueden ser menos agresivos

CON O SIN CRIBADO

4 habrán muerto de cáncer de mama a pesar de haber participado en el cribado

8 habrán sido diagnosticadas y sobrevivirán

Habrían sobrevivido aunque no se hubieran cribado

EFECTOS ADVERSOS DEL CRIBADO

2 serán diagnosticadas y tratadas de un cáncer de mama

Este cáncer nunca habría sido una amenaza para su vida

40 habrán necesitado pruebas adicionales

Posteriormente se descartará que hubieran tenido cáncer de mama (falsos positivos)

Por cada muerte evitada por el programa de cribado, 2 mujeres son diagnosticadas y tratadas de un cáncer que nunca hubiera puesto en riesgo su vida.

LA DETECCIÓN PRECOZ DEL CÁNCER DE MAMA

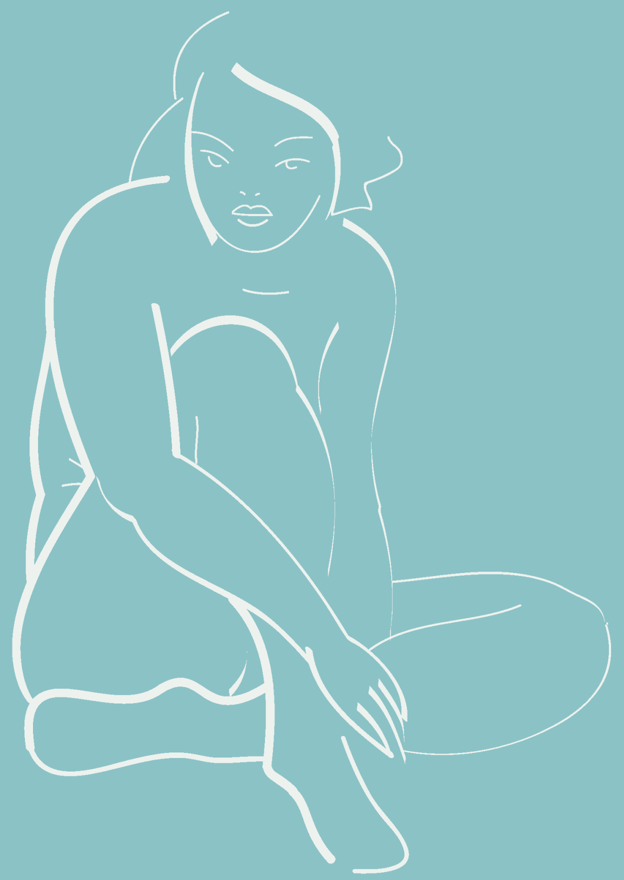

AYUDÁNDOTE A DECIDIR

PARTICIPAR O NO PARTICIPAR EN EL CRIBADO DEL CÁNCER DE MAMA: ESTA ES LA CUESTIÓN

Estudios científicos recientes han identificado efectos adversos, antes desconocidos, de la detección precoz de cáncer de mama mediante mamografía. Por esta razón, este folleto tiene como objetivo informar sobre los beneficios y efectos adversos de participar en la detección precoz de cáncer de mama.

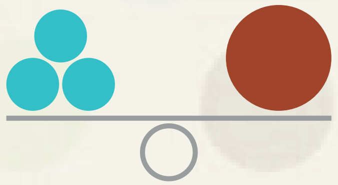

Este material informativo pretende ayudarte a sopesar pros y contras para que puedas tomar una decisión personal sobre si deseas participar o no en la detección precoz del cáncer de mama, en función de tus valores y preferencias.

¿QUÉ ES EL CÁNCER DE MAMA?

El cáncer de mama se desarrolla cuando algunas células empiezan a crecer de forma descontrolada, formando un tumor. A medida que el tumor crece las células malignas se pueden desplazar a otras partes del cuerpo y poner en peligro la vida de la persona afectada.

En Cataluña se diagnostican unos 4.000 casos nuevos de cáncer de mama al año. Las estadísticas nos dicen que 1 de cada 9 mujeres padecerá cáncer de mama a lo largo de su vida y que el 83% de las mujeres afectadas sobrevivirán a esta enfermedad.

¿QUÉ ES LA DETECCIÓN PRECOZ DEL CÁNCER DE MAMA?

La detección precoz del cáncer de mama, también denominada cribado, tiene por objetivo detectar un cáncer de mama en una etapa muy inicial, antes de que aparezcan síntomas. En su etapa inicial, el cáncer es más fácil de tratar y las oportunidades de sobrevivir son superiores.

El sistema sanitario público ofrece la posibilidad de participar en la detección precoz del cáncer de mama con el objetivo de reducir la mortalidad causada por este tumor. El programa de cribado se dirige a las mujeres entre 50 y 69 años y consiste en realizar una mamografía cada dos años.

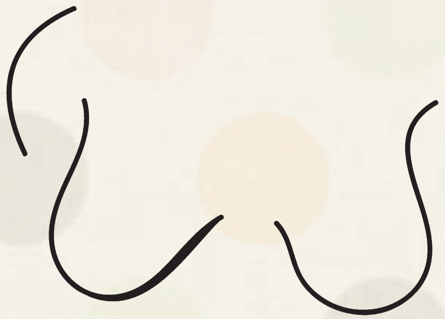

La mamografía es una radiografía de la mama. Es la prueba más eficaz para detectar el cáncer de mama en mujeres que no presentan síntomas. El riesgo de algún daño por la exposición a esta radiación es muy pequeño.

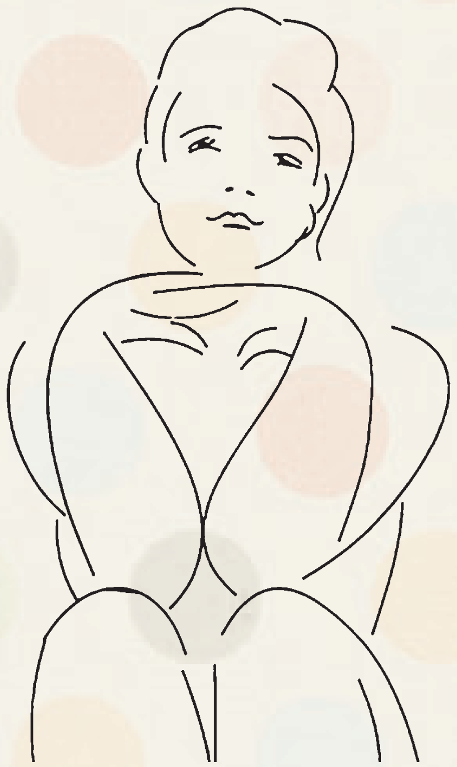

BENEFICIOS DEL CRIBADO

El cribado reduce el riesgo de morir por cáncer de mama

La detección precoz puede salvar la vida a algunas mujeres porque se diagnostican y tratan antes de lo que se habría hecho sin cribado.

De cada 200 mujeres que se realizan mamografías de cribado cada dos años, entre los 50 y los 69 años, 1 mujer se salva gracias a la detección precoz del tumor.

El cribado detecta el cáncer en estadios más iniciales

Un cáncer detectado en estadios iniciales no necesita tratamientos tan agresivos como cuando está más avanzado; estos tratamientos tienen menos efectos secundarios y la probabilidad de recuperación es más alta.

EFECTOS ADVERSOS DEL CRIBADO

Errores en el diagnóstico: falsos positivos y falsos negativos

Los falsos positivos se producen cuando los resultados de la mamografía hacen sospechar de un posible cáncer de mama que en realidad no existe. Esto conlleva exploraciones adicionales que no serían necesarias.

De cada 200 mujeres que se realizan mamografías de cribado cada dos años entre los 50 y los 69 años, 40 tendrán un resultado falso positivo.

La situación contraria, el falso negativo, es mucho menos frecuente y se puede producir cuando la mamografía no muestra ninguna señal de cáncer de mama, aunque la mujer lo padezca.

El cribado puede detectar tumores inofensivos

Algunos tipos de cáncer que se detectan mediante la mamografía de cribado crecen tan lentamente que nunca hubieran llegado a ser un problema de salud. Algunos, incluso, habrían desaparecido de forma espontánea, sin tratamiento.

Actualmente no se puede saber qué lesiones progresarían y cuáles no, y por tanto, se ofrece tratamiento a todas las mujeres diagnosticadas. Algunas mujeres pueden recibir tratamientos que tienen efectos secundarios importantes, sin necesitarlos. Esto se conoce como **sobrediagnóstico** y **sobretratamiento**.

De cada 200 mujeres que se realizan mamografías de cribado cada dos años, entre los 50 y los 69 años, 2 serán tratadas de cáncer sin necesidad.
