# Supplementary material for: The effect of information about the benefits and harms of mammography on women’s decision-making: study protocol for a randomized controlled trial
Source: Trials. 2017 Sep 12;18:426. doi: 10.1186/s13063-017-2161-7 (PMC5596499; doi:10.1186/s13063-017-2161-7)
Supplement: Supplementary file 4 — Post-intervention questionnaire (Q2). Study outcomes. (PDF 298 kb) [file 13063_2017_2161_MOESM4_ESM.pdf]

# Estudio InforMa. Cuestionario 2

El estudio **InforMa** es un trabajo de investigación en el que colaboran las Universidades de Lleida, Rovira i Virgili, el Instituto Hospital del Mar de Investigaciones Medicas (IMIM), el Instituto Catalán de Oncología y el Servicio Canario de Salud y que tiene por finalidad:

(A) Evaluar el impacto de una herramienta que ayude a la toma de decisiones compartidas en el proceso de participación en un programa de detección precoz de cáncer de mama.

(B) Evaluar las preferencias de las mujeres en relación a la adaptación del programa de detección precoz al riesgo individual de tener cáncer.

La participación al estudio comporta responder dos cuestionarios. Este es el segundo. La información recogida en el estudio será totalmente confidencial. Sus respuestas se introducirán en una base de datos protegida que imposibilitará la identificación mediante un sistema de disociación, de acuerdo con la Ley Orgánica 15/1999 de Protección de Datos de Carácter Personal.

**Cuando responda las preguntas de los cuestionarios no se preocupe por si las respuestas son correctas o incorrectas. ¡Piense que no está haciendo un examen!**

Si marca una **X** en el recuadro entendemos que da su consentimiento para participar en el estudio: ☐

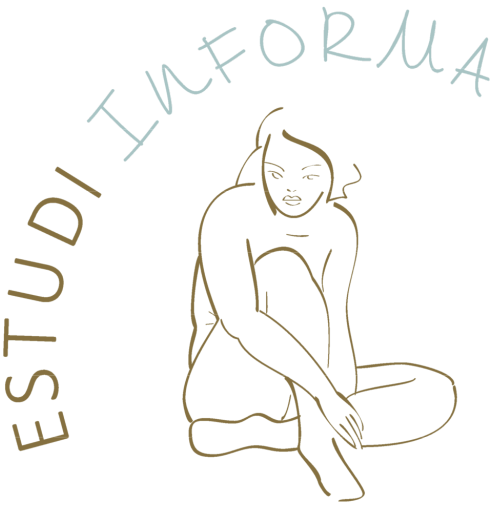

# 1. Valoración del folleto informativo

A continuación le haremos algunas preguntas sobre el folleto informativo que le hemos facilitado. Si no lo ha recibido, le agradeceríamos que se pusiera en contacto con nosotros (*estudi.informa@gmail.com*). ¡Gracias!

**1. ¿Ha leído todo el folleto?**
- ○ Sí
- ○ No

**2. Opina que el folleto es. . .**
- ○ Demasiado largo
- ○ Un poco largo
- ○ Correcto
- ○ Un poco corto
- ○ Demasiado corto

**3. La orientación del folleto le ha parecido. . .**
- ○ Claramente a favor del cribado
- ○ Más bien a favor del cribado
- ○ Equilibrado
- ○ Más bien en contra del cribado
- ○ Claramente en contra del cribado

**4. La información del folleto es clara y fácil de entender**
- ○ Totalmente de acuerdo
- ○ De acuerdo
- ○ Ni de acuerdo ni en desacuerdo
- ○ En desacuerdo
- ○ Muy en desacuerdo

**5. El folleto es muy útil para tomar una decisión sobre el cribado de cáncer de mama**
- ○ Totalmente de acuerdo
- ○ De acuerdo
- ○ Ni de acuerdo ni en desacuerdo
- ○ En desacuerdo
- ○ Muy en desacuerdo

# 2. Conocimientos

A continuación le haremos algunas preguntas sobre sus conocimientos sobre la mamografía y el cáncer de mama.

**6. ¿Qué es una mamografía de cribado?**
- ○ Una mamografía que se hace cuando no hay ningún síntoma
- ○ Una mamografía que se hace cuando se nota algún cambio o alteración en el pecho

**7. Usted cree que si hay un cáncer de mama, ¿la mamografía siempre lo detectará?**
- ○ Sí
- ○ No

**8. Todas las mujeres con un resultado de mamografía sospechoso, ¿tienen cáncer de mama?**
- ○ Sí
- ○ No

**9. ¿Quién cree que es más probable que muera de cáncer de mama?**
- ○ Una mujer que se hace mamografías de cribado
- ○ Una mujer que no se hace mamografías de cribado

**10. ¿Quién cree que tiene más posibilidades de ser diagnosticada de cáncer de mama?**
- ○ Una mujer que se hace mamografías de cribado
- ○ Una mujer que no se hace mamografías de cribado

**11. Todos los cánceres de mama resultarán mortales si no se diagnostican y tratan. Esta afirmación es:**
- ○ Cierta
- ○ Falsa

**12. Cuando el cribado detecta un cáncer, los médicos pueden predecir con certeza si acabará causando problemas de salud. Esta afirmación es:**
- ○ Cierta
- ○ Falsa

**13. A pesar de que hay cánceres de mama que no causarían problemas de salud, todos se tratan. Esta afirmación es:**
- ○ Cierta
- ○ Falsa

**14. El cribado hace que algunas mujeres con cánceres de bajo riesgo, que nunca ocasionarían ningún problema de salud, sean tratadas de manera innecesaria. Esta afirmación es:**
- ○ Cierta
- ○ Falsa

**15. Es más frecuente que el cribado detecte cánceres de bajo riesgo, que nunca ocasionarían ningún problema de salud, que no que evite la muerte por cáncer de mama. Esta afirmación es:**
- ○ Cierta
- ○ Falsa

**16. ¿Cuál de las dos frases siguientes describe mejor el sobrediagnóstico?**
- ○ El cribado detecta cánceres que nunca habrían causado problemas de salud
- ○ El cribado puede detectar anomalías, pero otras pruebas adicionales demuestran que no es cáncer

Para las siguientes preguntas, imagine 200 mujeres que se hacen mamografías de cribado cada 2 años desde los 50 hasta los 69 años. A lo largo de todo este tiempo:

**17. Algunas mujeres serán diagnosticadas de cáncer de mama. ¿Cuántas muertes por cáncer de mama se evitan gracias al cribado?**
- ○ 0
- ○ 1-2
- ○ 3
- ○ $>3$
- ○ No lo sé

**18. Algunas mujeres serán diagnosticadas de cáncer de mama. ¿Cuántas cree que morirán de cáncer de mama, a pesar de haber participado en el cribado?**

- ○ 0
- ○ 1
- ○ 2-6
- ○ 7-10
- ○ $>$ 10
- ○ No lo sé

**19. Algunas mujeres serán diagnosticadas de cáncer de mama. ¿Cuántas cree que serán diagnosticadas y tratadas de un cáncer de mama que nunca habría causado un problema de salud?**

- ○ 0
- ○ 1-4
- ○ 5
- ○ $>$ 5
- ○ No lo sé

**20. ¿Cuántas mujeres tendrán un resultado falso positivo? (Un falso positivo es un resultado sospechoso en la mamografía que las pruebas adicionales mostrarán que no es un cáncer.)**

- ○ $<$ 20
- ○ 20-29
- ○ 30-50
- ○ 51-60
- ○ $>$ 60
- ○ No lo sé

## 3. Actitudes

**21. Para usted hacerse mamografías de cribado:**

| | | |
|---|---|---|
| **Tiene pocos beneficios** | 1 ○—○—○—○—○ 5 | **Tiene muchos beneficios** |
| **Tiene muchos efectos adversos** | 1 ○—○—○—○—○ 5 | **Tiene pocos efectos adversos** |
| **No es una buena opción** | 1 ○—○—○—○—○ 5 | **Es muy buena opción** |
| **No es importante** | 1 ○—○—○—○—○ 5 | **Es muy importante** |
| **No vale la pena** | 1 ○—○—○—○—○ 5 | **Vale mucho la pena** |

## 4. Intenciones

**22. Tiene intención de participar en el programa de cribado o de hacerse mamografías de cribado fuera del programa?**

- ○ Seguro que sí
- ○ Probablemente sí
- ○ No estoy segura
- ○ Probablemente no
- ○ Seguro que no

## 5. Decisiones

**23. Respecto a su participación en el programa de cribado, cómo de segura se siente...**

| | 1. Sí | 2. No | 3. No está segura |
|---|---|---|---|
| **1. ¿Está segura de lo que hará?** | ○ | ○ | ○ |
| **2. ¿Tiene claro cuál es su mejor opción?** | ○ | ○ | ○ |

**24. Respecto a sus conocimientos sobre el cribado...**

| | 1. Sí | 2. No | 3. No está segura |
|---|---|---|---|
| **3. ¿Sabe qué opciones tiene?** | ○ | ○ | ○ |
| **4. ¿Sabe cuáles son las ventajas de cada opción?** | ○ | ○ | ○ |
| **5. ¿Sabe cuáles son las desventajas de cada opción?** | ○ | ○ | ○ |

**25. Respecto a lo que es importante para usted...**

| | 1. Sí | 2. No | 3. No está segura |
|---|---|---|---|
| **6. ¿Tiene claro cuáles son las ventajas más importantes para usted?** | ○ | ○ | ○ |
| **7. ¿Tiene claro cuáles son las desventajas más importantes para usted?** | ○ | ○ | ○ |

**26. Respecto de los apoyos con los que puede contar...**

| | 1. Sí | 2. No | 3. No está segura |
|---|---|---|---|
| **8. ¿Tiene suficiente apoyo para poder escoger una opción?** | ○ | ○ | ○ |
| **9. ¿Puede decidir sin presiones de nadie?** | ○ | ○ | ○ |
| **10. ¿Tiene suficientes asesoramiento para poder tomar una decisión?** | ○ | ○ | ○ |

## 6. Confianza en la decisión

En cuanto a su autoconfianza, valore las siguientes frases en una escala del 1 al 5 (1= muy poco y 5=mucho)

| | | |
|---|---|---|
| **27.** | **¿Cuán segura se siente de su decisión?** | Muy poco ○—○—○—○—○ Mucho |
| **28.** | **¿Cuán segura está de haber entendido la información para poder tomar una decisión?** | Muy poco ○—○—○—○—○ Mucho |
| **29.** | **¿Cuán segura está de haber llegado a la mejor decisión para usted?** | Muy poco ○—○—○—○—○ Mucho |

# 7. Estado emocional

**30. Valore cómo se siente en estos momentos. Conteste de forma intuitiva y tenga en cuenta que no hay respuestas erróneas. (1=nada; 4=mucho)**

| | 1. Nada | 2. Un poco | 3. Bastante | 4. Mucho |
|---|---|---|---|---|
| **1. Estoy tranquila** | ○ | ○ | ○ | ○ |
| **2. Estoy nerviosa** | ○ | ○ | ○ | ○ |
| **3. Estoy inquieta** | ○ | ○ | ○ | ○ |
| **4. Estoy relajada** | ○ | ○ | ○ | ○ |
| **5. Estoy contenta** | ○ | ○ | ○ | ○ |
| **6. Estoy preocupada** | ○ | ○ | ○ | ○ |

**31. ¿Está preocupada por el cáncer de mama?**

○ Nada o muy poco
○ Un poco
○ Bastante
○ Mucho

# 8. Valoración anticipada

**32. Si NO SE HACE mamografías de cribado, más adelante se arrepentirá**

○ Totalmente de acuerdo
○ De acuerdo
○ Ni de acuerdo ni en desacuerdo
○ En desacuerdo
○ Totalmente en desacuerdo

**33. Si SE HACE mamografías decribado, más adelante se arrepentirá**

○ Totalmente de acuerdo
○ De acuerdo
○ Ni de acuerdo ni en desacuerdo
○ En desacuerdo
○ Totalmente en desacuerdo

# 9. Perspectiva temporal

**34.**

| | 1. Totalmente de acuerdo | 2. De acuerdo | 3. Ni de acuerdo ni en desacuerdo | 4. En desacuerdo | 5. Totalmente en desacuerdo |
|---|---|---|---|---|---|
| **1. Piensa en el futuro y esto influye en su comportamiento actual** | ○ | ○ | ○ | ○ | ○ |
| **2. No piensa en lo que pueda pasar en el futuro** | ○ | ○ | ○ | ○ | ○ |
| **3. Está dispuesta a sacrificarse por estar mejor en el futuro.** | ○ | ○ | ○ | ○ | ○ |
| **4. Prefiere pensar en el presente más que en el futuro.** | ○ | ○ | ○ | ○ | ○ |

# 10. Importancia percibida de los beneficios / efectos adversos del cribado

**35. En el momento de tomar la decisión sobre participar o no en el cribado…**

| | 1. Muy importante | 2. Bastante importante | 3. Poco importante | 4. Nada importante |
|---|---|---|---|---|
| **1. Poder evitar la muerte por cáncer de mama es para usted** | ○ | ○ | ○ | ○ |
| **2. La posibilidad de ser sobrediagnosticada y tratada de un cáncer que nunca le hubiera dado problemas de salud es para usted** | ○ | ○ | ○ | ○ |
| **3. La posibilidad de tener un falso positivo y necesitar pruebas adicionales a la mamografía de cribado para descartar un cáncer de mama es para usted** | ○ | ○ | ○ | ○ |

# 11. Riesgo personal percibido de cáncer de mama

**36. El riesgo que cree que tiene usted de ser diagnosticada de cáncer de mama a lo largo de su vida es:**

○ Muy bajo

○ Bajo

○ Moderado

○ Alto

**37. El riesgo que cree que tiene usted de ser diagnosticada de cáncer de mama en relación al resto de mujeres es:**

○ Mucho más bajo
○ Un poco más bajo
○ El mismo
○ Un poco más alto
○ Mucho más alto

## 12. Probabilidad percibida respecto a las mujeres que se hacen mamografías de cribado

**38. Si usted se hace mamografías de cribado, cree que su probabilidad de evitar la muerte por cáncer de mama, respecto al resto de mujeres, es**

○ Mucho más baja
○ Un poco más baja
○ La misma
○ Un poco más alta
○ Mucho más alta

**39. Si usted se hace mamografías de cribado, cree que su probabilidad de ser diagnosticada y tratada de un cáncer de mama que nunca le hubiera provocado un problema de salud (sobrediagnóstico), respecto al resto de mujeres, es**

○ Mucho más baja
○ Un poco más baja
○ La misma
○ Un poco más alta
○ Mucho más alta

**40. Si usted se hace mamografías de cribado, cree que su probabilidad de necesitar pruebas adicionales a la mamografía para descartar un cáncer de mama (Falso positivo), respecto al resto de mujeres, es**

○ Mucho más baja
○ Un poco más baja
○ La misma
○ Un poco más alta
○ Mucho más alta

**41. En el recuadro siguiente puede escribir los comentarios o dudas que tenga sobre el estudio o las preguntas del cuestionario.**

¡Muchas gracias por su colaboración!

GOBIERNO DE ESPAÑA
MINISTERIO DE ECONOMÍA Y COMPETITIVIDAD
Instituto de Salud Carlos III
Unión Europea
Fondo Europeo de Desarrollo Regional
*"Una manera de hacer Europa"*
